# Perinatal Mortality and Associated Factors Among Antenatal Care Attended Pregnant Mothers at Public Hospitals in Gamo Zone, Southern Ethiopia

**DOI:** 10.3389/fped.2020.586747

**Published:** 2020-12-23

**Authors:** Samuel Dessu, Zinabu Dawit

**Affiliations:** ^1^Department of Public Health, College of Medicine and Health Sciences, Wolkite University, Wolkite, Ethiopia; ^2^Department of Nursing, Arba Minch Health Science College, Arba Minch, Ethiopia

**Keywords:** magnitude, perinatal mortality, public hospitals, Southern Ethiopia, Gamo Zone

## Abstract

**Introduction:** Perinatal mortality is the death of a baby between 28 weeks of gestation onwards and before the first 7 days of life. According to WHO, Ethiopia is one of the most commonly noticed country in the world in considering perinatal mortality rate. The overall perinatal mortality rate in Ethiopia was around 66–124 per 1,000 births.

**Objective:** To determine the magnitude of perinatal mortality and associated factors among mothers who attended antenatal care at public hospitals in Gamo Zone, Southern Ethiopia.

**Methods:** A cross-sectional study was conducted at Arba Minch General Hospital and Chencha District Hospital antenatal care attended by pregnant mothers from the 1st of February to the 28th of March 2019, among the mothers enrolled at ANC clinic from the 1st of January to the 30th of December 2018 using a simple random sampling method for the pre-determined 1,820 records. Both bivariate and multivariable logistic regression analysis was conducted. Variables which had a *p*-value <0.25 in bivariate analysis were considered as a candidate variable for multivariable analysis and variables which had a *P*-value <0.05 in multivariable analysis were declared as statically significant.

**Results:** The prevalence of perinatal mortality was 12.6% (95% CI: 11.80, 13.40) and grand multiparity (AOR: 7.40; 95% CI: 2.77, 20.26), having one antenatal visit (AOR: 4.40; 95% CI: 1.64, 11.91), spontaneous vaginal delivery (AOR: 0.36; 95% CI: 0.16, 0.82), being pre-term (AOR: 6.78; 95% CI: 2.41, 19.09), birth weight <2,500 gram (AOR: 3.10; 95% CI: 1.48, 6.46), maternal ever hemoglobin level <10 gm/dl (AOR: 4.04; 95% CI: 1.91, 8.57), and pre-partum onset of pregnancy induced hypertension (AOR: 4.01; 95% CI: 2.01, 6.08) were statistically significant in the multivariable logistic regression model.

**Conclusion:** The magnitude of perinatal mortality was high as compared with the Ethiopian Health and Demographic Survey report 2016 and high parity, low in number of antenatal care visits, low gestational age, low birth weight, low maternal hemoglobin level, and pre-partum onset of pregnancy induced hypertension were independent factors which increase the perinatal mortality while spontaneous vaginal delivery reduces the mortality risk. Therefore; the community should be educated to reduce the number of instance of births. In addition; the health care professionals should emphasize on the care provided for the newborns having low birth weight and use spontaneous vaginal delivery as much as possible.

## Introduction

Availability and quality of healthcare of both mother and newborn is reflected in the perinatal mortality rate. Perinatal mortality remains one of the devastating pregnancy outcomes for millions of families in low-and-middle-income countries (1).

Perinatal mortality rates play an increasingly important role in childhood mortality, and there are currently no effective institution and community-based intervention programs in Ethiopia particularly targeting perinatal mortality including stillbirth (2). Perinatal mortality rate (PMR) is taken as one of the indicators of the health status of a given society. It is multifactorial in etiology and depends on the quality of health care provided to the pregnant women and their babies (3).

According to the World Health Organization (WHO), perinatal mortality is the death of a baby between 28 weeks of gestation onwards and before the first 7 days of life (4). The perinatal mortality rate (PMR) is calculated by incorporating all stillbirths and early neonatal deaths in a specified period per the total number of births multiplied by thousand (5, 6).

The 2010 global health estimate indicated that there were around 2.6 million still births at the end of 2010. With this still births, half of them were occur during labor and delivery. In addition, around 2.8 million newborn deaths were occurring in the 1st week of life. More than half of these new born deaths were in the low and middle income countries (7). Globally, nearly 8,000,000 perinatal deaths are reported in a single year. Around 40–60% of this perinatal mortality is occurring within the first 7 days of life and almost all of this death is found in developing countries (8). The perinatal mortality rate is extremely different between developing countries (50 per 1,000 total births) and developed countries (10 per 1,000 total births) (4).

In developing countries, labor onset, mode of delivery, gestational age, pregnancy induced hypertension, and low in number of antenatal care (ANC) visits are the commonly mentioned factors which increase the risk of perinatal mortality (9, 10). In addition; maternal hemoglobin level and provision of anticonvulsant and antihypertensive were the commonly contributing factors for perinatal mortality (11, 12).

According to the World Health Organization estimate, Ethiopia is one of the most leading countries having high perinatal mortality (4). The perinatal mortality rate in Ethiopia was around 16.5 per 1,000 births (13). The rate of stillbirths based on the facility and community based studies was 60–110 per 1,000 births and the rate of early neonatal deaths was 20–34/1,000 births (13).

In Ethiopia, since there is a high number of a new born death, only little is known about the magnitude of perinatal mortality and associated factors. Therefore, this study was conducted to estimate the magnitude of perinatal mortality and associated factors among mothers attending antenatal care at Gamo Zone, Southern Ethiopia, which will be an input for the governmental and non-governmental stakeholders and for the researchers as a base line study.

## Methods

A cross sectional study was employed among ANC attendant pregnant mothers at public hospitals in Gamo Zone, Southern Ethiopia specifically at the Arba Minch General Hospital and Chencha District Hospital. These two hospitals were the only hospitals which provide antenatal care service, delivery service, and neonatal intensive care unit service at the study period within the Gamo Zone. There is a project working in the area (Arba Minch Health and demographic surveillance system, AM-HDSS). This project works in nine Kebelles in Arba Minch Zuria Woreda.

The study was conducted from the 1st of February to the 28th of March 2019 among the mothers enrolled at ANC clinics from the 1st of January to the 30th of December 2018. All maternal-perinatal pairs who have ANC visits at Gamo Zone Public Hospitals were the source populations and the study populations were all the selected maternal-perinatal pairs who have ANC visits at Gamo Zone public hospitals.

Simple random sampling method using computer generated random numbers was employed for the predetermined 1,821 samples (which was calculated using double population proportion formula for the factors in considering the assumptions, 95%CI, power: 80, exposed to non-exposed ratio: 2, proportion of outcome among non-exposed: 51%, proportion of outcome among exposed: 58.06 and adjusted odd ratio for the variable gestational age <34 weeks: 1.33) (9). Sample size was allocated to both hospitals proportionally based on the number of antenatal records of mothers.

The dependent variable was a perinatal death (defined as the sum of still birth or fetal deaths in pregnancies >28 weeks of gestation and early neonatal mortality or deaths within the 1st weeks of life) and the independent variables were categorized under maternal, obstetric related factors, and pregnancy induced hypertension, & related factors.

**Figure 1:**
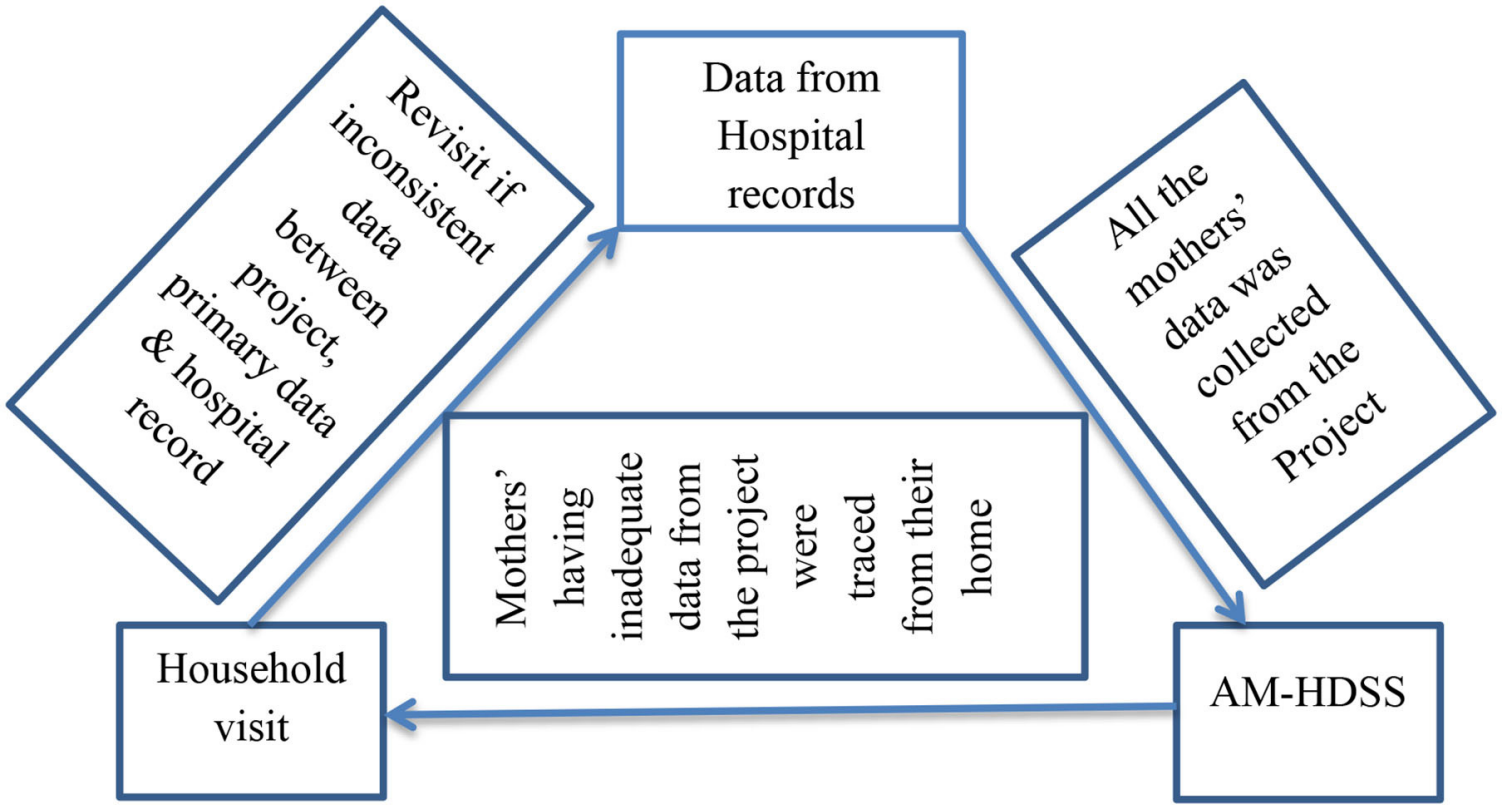
Information flow system framework among ANC attended pregnant mothers at Gamo Zone public hospitals.

The source of data was both individual records of the mothers and the ANC attended mothers themselves. Initially, the records of the mothers were abstracted from the record room at the Arba Minch General Hospital and Chencha district Hospital using their ANC register form at a sampling frame and all relevant information were collected, then using their information from ANC, delivery and postnatal register their data were traced and collected from the Arba Minch Health and demographic surveillance (AM-HDSS). Those mothers having incomplete data from the project were traced in their address and a direct interview was made with the mothers and all the relevant information was collected. Additionally; for the mothers having inconsistent data, again, a hospital record revisit was made (Figure 1).

Data were collected by using a structured checklist which was prepared in English. Data entry, coding, editing, and cleaning were performed using the Epi Data version 3.5.1 and exported to the Stata version 14 for analysis. Both Bivariate and multivariable logistic regression analyses was conducted to determine the associations between dependent and the explanatory variables. Crude odd ratio (COR), adjusted odd ratio (AOR), 95% confidence interval (CI), and *P*-value were used to assess the strength of association and statistical significance.

Data cleaning was performed by checking for frequencies, accuracy, outliers, and consistencies and missed values, and variables. Frequencies, proportions, and summary statistics were used to describe the study population about relevant variables using tables, charts, and graphs. Variables which had a *p*-value <0.25 in the bivariate analysis were employed as a candidate variable for the multivariable logistic regression model and the variables which had a *P*-value <0.05 in the multivariable analysis were considered as statically significant.

Ethical clearance was obtained from the Arba Minch Health Science College ethical review board. In addition; permission letter was obtained from the Arba Minch Health Science College, Gamo Zone Health Department and the corresponding hospital administrators. An informed consent was made by the mothers. The hospital administrators were informed about the study and they gave a permission to use the record based on the letter obtained from the Zonal Health department. All precautions were undertaken to protect the confidentiality of their personal information through using a pass word for the data and removing names of study subjects in the data extraction tool.

## Results

### Socio-Demographic Characteristics

A total of 1,820 study participants were involved—a response rate of 99.90%. The minimum and maximum age of the mothers was 16 years and 39 years old, respectively, with the mean age of 25.87 ± 4.72 years old. The number of mothers who had age <19 years old and 20–34 years old were 86 (4.70%) and 1,609 (88.40%), respectively. Regarding their residence, majority of the mothers (80.2%) were urban residents.

### Obstetric Characteristics

From the total clients, nearly one fourths (27.30%) were primigravidas, while multigravidas (I–IV births) and grand multiparas (five and above births) accounted for 64.50 and 8.20% of the mothers, respectively. Among the mothers, 862 (47.40%) of them attended one ANC visit and 235 (12.90%) attended four and above visits. Majority of the births (96.60%) of them were single tone pregnancies. The gestational age was ranged from 29 to 42 weeks, with the mean of 36.65 ± 2.28 weeks. Similarly the minimum and maximum birth weight of the baby was 600 gram and 5,500 gram, respectively, with the mean of 3,187 ± 661.37 gram. Among them, only 1,584 (87.03%) of them were measured their weights (Table 1).

**Table 1:** Obstetric characteristics of perinatal mortality among mothers who attended ANC in Gamo Zone, Southern Ethiopia, 2019.

**Variable**	**Category**	**No(*n*)**	**Percent(%)**
Number of antenatal care visits	One	862	47.40
	Two	446	24.50
	Three	277	15.20
	Four and above	235	12.90
Labor onset	Spontaneous	1,573	86.40
	Induced	162	8.90
	Direct cesarean section	85	4.70
Mode of delivery	Spontaneous vaginal delivery	1,488	81.80
	Instrumental delivery	112	6.20
	Elective cesarean section	220	12.10
Gestational age(weeks)	<34	354	19.50
	34–36	507	27.90
	>37	959	52.70
Birth weight in gram (*n* = 1,584)	<2,500	215	13.57
	>2,500	1,369	86.43

### Pregnancy Induced Hypertension and Related Characteristics

From the total respondents, nearly two-thirds (67.80%) of the mothers did not face pregnancy induced hypertension. From those who had pregnancy induced hypertension, prepartum, intrapartum, and postpartum onset accounted 480 (81.90%), 71 (12.13%), and 35 (5.97%), respectively. The mean of ever noticed recorded systolic blood pressure level was 119.1 (±19.50) mmHg with the highest and lowest level of 185 and 75 mmHg, respectively, while it was 79.6 (±13.60) mmHg for ever noticed recorded diastolic blood pressure level with the highest and lowest level of 125 and 44 mmHg, respectively.

Regarding the type of pregnancy induced hypertension, 304 (51.90%) of them had severe preeclampsia. The minimum and maximum hemoglobin level of the mothers was 7 g/dl and 16 g/dl, respectively, with the mean of 12.40 ± 1.40 g/dl. In addition, 265 (14.60%) of the mothers had hemoglobin level <10 g/dl (Table 2).

**Table 2:** Pregnancy induced hypertension and related characteristics of perinatal mortality among mothers who attended ANC at Gamo Zone, Southern Ethiopia, 2019.

**Variables**	**Category**	**Frequency(*n*)**	**Percent(%)**
Highest SBP (mmHg)	<140	1,610	88.50
	141–159	109	6.00
	>160	101	5.50
Highest DBP(mmHg)	<90	1,620	89.00
	91–110	141	7.70
	>110	59	3.20
Type of pregnancy induced hypertension (*n* = 586)	Severe preeclampsia	304	51.90
	Eclampsia	186	31.70
	HELLP syndrome	96	16.40
Anticonvulsant given(*n* = 586)	Yes	340	58.00
	No	246	42.00
Type of anticonvulsant (*n* = 340)	MgSO_4_	273	80.30
	Diazepam	67	19.70
Antihypertensive given (*n* = 586)	Yes	298	50.80
	No	288	49.20
Maternal ever hemoglobin level during pregnancy (gm/dl)	<10	265	14.60
	10–11.9	371	20.40
	>12	1,184	65.10

### Magnitude of Perinatal Mortality

Among the total 1,820 respondents, 230 of perinates were dead. Therefore, the magnitude of perinatal mortality was 12.60% (95%CI: 11.80, 13.40). From the dead perinates, 135 (58.69%) were dead before 34 weeks of gestation while 77 (33.48%) were at 34–36 weeks of gestation. The remaining 12(5.22%) were dead after 36 weeks of gestation which was stillbirth and 6 (2.61%) were dead at early neonatal periods. Nearly two fifths (36%) of the dead perinates were delivered from mothers who had pregnancy induced hypertension.

### Factors Associated With Perinatal Mortality

To identify potential factors associated with perinatal mortality, binary logistic regression model was run independently for perinatal, maternal, and obstetrical and pregnancy induced hypertension related factors. In bivariate analysis, place of residence, parity, number of ANC visits, labor onset, mode of delivery, gestational age, birth weight, onset of pregnancy induced hypertension, and maternal hemoglobin level were the candidate variables for multivariable analysis; and parity, mode of delivery, gestational age, birth weight, and maternal hemoglobin level were factors which are statistically significant in multivariable analysis.

Mothers who had five and above instance of birth had seven times more likely to have perinatal mortality as compared with those of primigravidas (AOR: 7.40; 95%CI: 2.77, 20.26). Mother who had attended only one antenatal visit had four times more likely to have perinatal mortality as compared with those of mothers who had four and above antenatal visits (AOR: 4.40; 95%CI: 1.64, 11.91). Perinates who were delivered by spontaneous vaginal delivery were 64% less likely to have risk of perinatal mortality as compared with those delivered by elective cesarean section (AOR: 0.36; 95%CI: 0.16, 0.82). The odd of perinatal mortality among perinates delivered by assisted instrumental delivery were five times more likely as compared with those delivered by elective cesarean section (AOR: 5.63; 95%CI: 1.58, 20.14). Preterm newborns were more at risk for perinatal mortality than the counterparts >37 weeks and above gestational age (AOR: 6.78; 95%CI: 2.41, 19.09).

Perinates having a birth weight <2,500 gm had three times more likely to die as compared with those who had birth weight more than 2,500 gm (AOR: 3.10; 95%CI: 1.48, 6.46). The likelihood of perinatal mortality among mothers who had a hemoglobin level <10 and 10–11.9 gm/dl was four times more likely as compared with the counterparts who had 12 gm/dl and above [(AOR: 3.90; 95%CI: 1.79, 8.47) and (AOR: 4.04(95%CI: 1.91, 8.57), respectively]. Mothers who had prepartum onset of pregnancy induced hypertension had four times more likely to have perinatal mortality as compared with those of postpartum onset (AOR: 4.01; 95%CI: 2.01, 6.08) (Table 3).

**Table 3:** Factors associated with perinatal mortality among mothers who attended ANC in Gamo Zone public Hospitals, Southern Ethiopia, 2019.

**Variables**	**Category**	**Total**	**Death[*n*(%)]**	**COR(95%CI)**	**AOR(95%CI)**
Place of residence	Urban	1,460	163(11.20%)	1	1
	Rural	360	67(18.60%)	1.82(1.33,2.48)^*^	1.10(0.47, 2.38)
Parity	Primigravida	496	41(8.30%)	1	1
	Multigravida(I–IV)	1,173	142(12.10%)	1.53(1.06,2.20)^*^	1.98(0.92, 4.27)
	Grand multipara (>V)	151	47(31.10%)	5.02(3.14,8.02)^*^	7.40(2.77, 20.26)^**^
Number of ANC visits	One	235	38(16.20%)	1.43(0.95, 2.15)^*^	4.40(1.64, 11.91)^**^
	Two	277	26(9.40%)	0.77(0.49, 1.21)	1.40(0.50, 4.27)
	Three	446	64(14.30%)	1.24(0.89, 1.74)^*^	1.50(0.68, 3.38)
	Four and above	862	102(11.80%)	1	1
Labor onset	Spontaneous	1,573	186(9.92%)	1	1
	Induced	162	38(23.46%)	1.70(1.12, 2.62)^*^	0.29(0.07, 1.21)
	Direct CS	85	6(7.06%)	1.75(0.99, 3.08)^*^	1.62(0.26, 9.96)
Mode of delivery	SVD	1,488	84(5.65%)	7.38(4.57, 11.92)^*^	0.36(0.16, 0.820^**^
	Instrumental delivery	112	98(87.50%)	4.90(2.93, 8.21)^*^	5.63(1.58, 20.14)^**^
	Elective CS	220	48(21.82%)	1	1
Gestational age (weeks)	<36	861	134(15.56%)	32.20(19.28, 53.84)^*^	6.78(2.41, 19.09)^**^
	Above 36	959	96(10.01%)	1	1
Birth weight (gram)	<2,500	215	69(32.09%)	7.05(4.43, 11.21)^*^	3.10(1.48, 6.46)^**^
	>2,500	1,369	161(11.06%)	1	1
Maternal ever hemoglobin level during pregnancy (gm/dl)	<10	265	96(36.20%)	11.40(7.92, 16.5)^*^	4.04(1.91, 8.57)^**^
	10–11.9	371	78(21.00%)	5.36(3.72, 7.73)^*^	3.90(1.79, 8.47)^**^
	>12	1,184	56(4.70%)	1	1
Onset of pregnancy induced hypertension	Pre-partum	35	6(17.10%)	3.13(1.28, 7.71)^*^	4.01(2.01, 6.08)^**^
	Intra-partum	480	189(39.40%)	1.40(0.49, 3.98)	2.51(0.83, 6.02)
	Post-partum	71	16(22.50%)	1	1

* *Indicates candidate variables in bivariate analysis*.

** *Indicates statistically significant variables in multivariable analysis*.

## Discussion

This study revealed that the magnitude of perinatal mortality was 12.60%. This study finding was greater than the study conducted at the Kassala, Eastern Sudan (7.70%) and Jimma University Specialized hospital (9.83%) (14, 15). In addition; it was less than the study conducted at the Wolayita Soddo referral hospital (17.30%) and in West Gojjam (27.10%) (16, 17). This difference might be due to the variation of the government implementation concern across time variation and the variation of the government concern across countries. In addition; this might be due to the health institution related factors and facility setup such as absence of adequate infrastructures at neonatal ward, sub optimal obstetric care during antepartum, intra-partum, postpartum, and delay visit of the client toward the health institution as well as the delay initiation of health care by service provider.

Health facility based deliveries will overestimate perinatal mortality because those deliveries did not represent deliveries outside health facility (4). Most commonly, laboring mothers who visit hospitals are referred from the nearby primary health care facilities for better care, especially the condition of the mother as well as the newborn is subjected for referral. This referral system may take certain time until reaching the hospital, which increases the delay before reaching hospital. So, these conditions may indicate the difference in the magnitude of perinatal mortality across different hospital based studies.

The odds of perinatal mortality among mothers who have one antenatal visit were four times more likely as compared with those who had four and above visits (AOR: 4.40; 95%CI: 1.64, 11.91). This study finding is inconsistent with the study conducted at the Hawassa university hospital and Kassala, Eastern Sudan (14, 18). In addition, this finding is consistent with the study conducted at the Wolaita Soddo referral hospital, Southern Ethiopia and Jimma University Specialized Hospital, South West Ethiopia (15, 17). This indicates the beneficial effect of antenatal care in which mothers early recognize the health problems and pregnancy related complications.

Consistent with a study conducted at Hawassa university hospital; this study revealed that, mothers delivered by spontaneous vaginal delivery were 64% less likely to have perinatal mortality as compared with those mothers delivered by cesarean section (18). In addition; this study finding is inconsistent with the study conducted at Addis Ababa Ethiopia (19). A study conducted at the Marondera District, East of Zimbabwe also showed that normal spontaneous vaginal delivery is protective for perinatal death as compared with instrumental delivery of cesarean section (20). This might be due to the reason that cesarean section is recommended to perform if the mother develops obstetric complications.

Inconsistent with a study conducted at the Hawassa university hospital, Netherland and West Gojam; the likelihood of perinatal mortality among women with five and above instance of live births was seven times more likely as compared with those who had one instance of live birth (16, 18, 20). In addition; this study finding is consistent with the study finding conducted at Kassala, Eastern Sudan (17). The differences in finding of the studies might be due to the differences in the design of the study, difference in the study populations, and sample size.

In line with a study conducted at Hawassa university hospitals, North Shoa Zone Oromia region, West Gojam and Addis Ababa, Ethiopia; this study indicates that preterms were seven times more likely to die as compared with the terms (16, 18, 19, 21). This might be due to that preterm birth is related with their anatomical structure immaturity and physiological adjustment of all the systems of the newborn which may increase the perinatal mortality risk. Respiratory system immaturity, respiratory distress syndrome (RDS), and susceptibility to certain infections due to the undeveloped immune system are the most common causes of preterm death (22).

Consistent with a study conducted at the Hawassa university hospital, Wolaita Soddo referral hospital, Southern Ethiopia, Jimma University specialized hospital, South West Ethiopia, and Addis Ababa, Ethiopia; this study revealed that babies born with a birth weight <2,500 gm were three times more likely to die as compared with babies having a birth weight of 2,500 gm and above (13, 15, 17, 18). This might be due to the weight of newborns is related with the gestational age.

In line with a study conducted at Addis Ababa, Ethiopia; the likelihood of perinatal mortality among women who have ever hemoglobin level during pregnancy is three times greater than those who had 12 gm/dl and above (23). This might be due to the reason that anemia can increase maternal morbidity and poor pregnancy outcome (24).

The limitation of this study was, since the study was conducted through record review, some variables related with laboratory tests, socio economic, socio-demographic, and service related factors were missed. In addition, studies conducted on record missed the exact time when the perinates were died. In another sense, the health care quality was not assessed. Similarly, there may be a misclassification of study subjects in their hemoglobin level and gestational age.

## Conclusion

The magnitude of perinatal mortality was high as compared with the Ethiopian Health and Demographic Survey report 2016, and high parity, low in number of antenatal care visits, low gestational age, low birth weight, low maternal hemoglobin level, and prepartum onset of pregnancy induced hypertension were independent factors which increase the perinatal mortality while spontaneous vaginal delivery reduces the mortality risk. Therefore; the community should be educated to reduce the number of instance of births. In addition, the health care professionals should emphasize on the care provided for the newborns having low birth weight and use spontaneous vaginal delivery as much as possible.

## Data Availability Statement

The datasets used and analyzed during the current study will be made available by the corresponding author upon reasonable request.

## Ethics Statement

Ethical clearance was obtained from Arba Minch Health Science College ethical review board. In addition; permission letter was obtained from the Arba Minch Health Science College, Gamo Gofa zone health Bureau and the corresponding hospital administrators.

## Author Contributions

Both authors made a significant contribution to the work reported in the conception, study design, execution, acquisition of data, analysis and interpretation, took part in drafting the article, or critically reviewing the article for important intellectual content, gave final approval of the version to be published, have agreed on the journal to which the article has been submitted, and agree to be accountable for all aspects of the work.

## Conflict of Interest

The authors declare that the research was conducted in the absence of any commercial or financial relationships that could be construed as a potential conflict of interest.
